# Extensive Abdominal Wound Caused by a Bull’s Horn

**Published:** 1875-10

**Authors:** W. A. Carmichael

**Affiliations:** Loveland, O.


### EXTENSIVE ABDOMINAL WOUND CAUSED BY A BULL'S HORN.

By W. A. CARMICHAEL, M.D., Loveland, O.

May 27, 1873. I was called to visit Mr. Joseph Branch, of Branch Hill, Clermont county, Ohio, aged 72 years; occupation, farmer. One hour previous to my seeing him he had been gored by a bull, the horn having penetrated the right side, below the margin of the ribs, producing a transverse opening seven inches long, and entering into the cavity of the abdomen. Through this lacerated wound a portion of the bowels protruded, which were gathered up from the ground by Mr. Branch, as he stood erect, and by him replaced in the cavity of the abdomen. Having some distance to go before reaching his home, and while being conveyed there, his bowels protruded, and were replaced five times by those caring for him.

On my arrival at the bedside of Mr. Branch, I found him suffering from the shock to his nervous system, and from hæmorrhage, yet his mind was composed and firm as a general on the battle field. After removing some bloody clothes from the side, the intestines again escaped, or a portion of them, consisting of the ascending colon and omentum. After cleansing them of all dust and dirt, I once more returned them to the cavity of the abdomen, closing the opening with sutures and adhesive plasters, and then, applying the water-dressing compress and bandage, gave an opiate from which rest was procured.

Wednesday, May 28th. Reaction fully established; good night's rest; no fever. Continued same treatment.

Thursday, 29th. Patient rested well; no fever; ordered cathartic.

Friday, 30th. Had a good night's rest; cathartic acted freely; changed plasters; continued water dressing, with solution of carbolic acid to cloths.

Saturday, 31st. No material change; pulse natural; no inflammation; some fullness at seat of injury; treatment continued.

Sunday, June 1st. Removed dressings and a portion of the sutures; some little suppuration.

Monday, 2d. Removed the remaining sutures; continued support, with the adhesive strips, carbolic ointment, compress and bandage; bowels move regularly; the fullness remains in side, but no inflammation; some soreness.

Wednesday, 4th. Patient continues to do well; thinks of plowing corn if no danger; advised to keep quiet.

Saturday, 7th. Removed dressings this day, and find wound completely closed, and no suppuration, this being just twelve days from the time of injury. Thus I dismiss my patient in a manner well, without having suffered from fever, and having had very little inflammation, and that only at the external wound.

This is one case, if not the only one on record, where such extensive injury has occurred with so little constitutional disturbance, and terminating favorably and completely in twelve days. The recovery of a patient, 72 years of age, after such an injury, makes this case one of peculiar interest to the profession.

July 20th, 1875. I this day saw Mr. Joseph Branch, who is now enjoying better health than he did previous to his injury, from which he has had no trouble or inconvenience the past two years, nor has he any at this time.

